# Life Cycle Impact Assessment of Garbage-Classification Based Municipal Solid Waste Management Systems: A Comparative Case Study in China

**DOI:** 10.3390/ijerph17155310

**Published:** 2020-07-23

**Authors:** Yujun Yuan, Tong Li, Qiang Zhai

**Affiliations:** Department of Mechanical Engineering, School of Mechanical, Electrical & Information Engineering, Shandong University, Weihai 264209, Shandong, China; 201700800324@mail.sdu.edu.cn (Y.Y.); 201600800288@mail.sdu.edu.cn (T.L.)

**Keywords:** municipal solid waste, life cycle impact assessment, garbage classification, uncertainty analysis

## Abstract

Confronted with a series of problems caused by surging generation of municipal solid waste (MSW), the Chinese central and local governments have promulgated and implemented policies to deal with them, including promotions of the classification of MSW. However, to date, practical knowledge and understanding about benefits for garbage classification from its environmental performance perspective is still limited. The present study is purposed to comprehensively investigate the environmental effects of garbage classification on municipal solid waste management (MSWM) systems based on three proposed garbage classification scenarios in China, via a comparative life cycle impact assessment (LCIA). Taking advantage of Impact Assessment of Chemical Toxics (IMPACT) 2002+ method, this comparative LCIA study can quantitatively evaluate midpoint, endpoint, and single scored life cycle impacts for the studied MSWM systems. A Monte Carlo uncertainty analysis is carried out to test the effectiveness and reliabilities of the LCIA results. The LCIA and uncertainty analysis results show that MSWM systems based on various garbage classification scenarios have significant variations in the studied midpoint, endpoint, and single scored environmental impacts. Different garbage classification scenarios have their individual environmental-friendly superiority for specific impact categories. Overall, results of this study demonstrate that MSW treatment systems integrated with garbage classification are more environmentally friendly by comparison with non-classification; and that the more elaborate the level of MSW classification, the smaller its impacts on the environment.

## 1. Introduction

### 1.1. Current Garbage Classification Practice

There are various practical approaches of garbage classification/sorting, in different countries. For instance, Japanese citizens are required to dispose garbage per local garbage sorting guidance, with specific requirements for disposal of incinerable and non-incinerable wastes. Germany adopts recyclable, nonrecyclable, and biomass wastes and with a specific focus on mechanical sorting and processing. The US puts efforts on waste source reduction and pays more attention on the recycling of paper, glass, plastics etc. Recent decades have witnessed the rapid increasing generation amount of China’s annual national municipal solid waste (MSW), from 0.025 billion tons in 1979 to 0.204 billion tons by approximate 7.12 times [1,2]. Aware of the severity of environmental impacts from MSW treatment, the central and local Chinese governments have been developing strategical and tactical policies, laws and regulations, to improve the environmental performances of the municipal solid waste management (MSWM) systems. For instance, Regulations on The Management of Domestic Garbage in Shanghai will be officially implemented as a mandatory garbage classification in Shanghai, and China plans to establish a nationwide garbage classification and processing system in 46 domestic cities [3]. As of the time of writing this paper, over 40 Chinese cities have set up pilot zones to promote garbage sorting and recycling [4,5,6,7,8,9,10,11]. However, effective promotion of the garbage classification is a complicated issue, involving economical topics such as charge mechanism and cost benefit, social topics such as behavior and psychology of residents, and of course the environmental issues [12].

### 1.2. State of the Art of Life Cycle Assessment on Municipal Solid Waste Management Systems

Life cycle assessment (LCA) has been widely applied as a systematical tool to evaluate the environmental performance of MSWM systems.

LCAs on MSWM technologies: LCA is used for the evaluation of MSWM technologies, such as fuel generation form MSW [13], valorizing MSW to bioenergy microbial protein, lactic and succinic acid via different biorefinery platforms [14], pyrolysis–gasification treatment processing of MSW [15], fast pyrolysis of MSW [16], use of compost from contaminated biodegradable MSW with silver and titanium dioxide nanoparticles [17], as well as comparisons in between practical technologies [18]. LCA has also been applied for assessing emerging new treatment technologies, such as an innovative and enhanced mechanical and biological treatment (MBT) demo plant installed in Mertesdorf (Germany), as part of Material Advanced Recovery Sustainable Systems [19], comparisons of three dual-stage advanced energy-from-waste technologies, i.e., gasification and plasma gas cleaning, fast pyrolysis and combustion and gasification with syngas combustion [20], mechanical biological pre-treatment of MSW [21]. Effects of specific treatment processing was also studied through LCA, such as street sweeping services [22], and source-separated collection [23]. LCA was also applied to investigate environmental burdens perceived through specific MSW processing, e.g., incineration [12,24,25,26], specifically, to quantitatively evaluate the life cycle environmental impacts of treatment of bottom ash from incineration [27,28,29].

MSW LCA case studies and scenario analysis: Many case studies have been conducted based on different geographical distributed MSWM systems, such as systems based in China [23,30], Sao Paulo, Brazil [31], Finland and China comparison [32], Sakarya, [33], Naples, Italy [34], Lahore, Pakistan [35], Bolivia [36], and France [37]. Scenario analysis is a commonly used method in the conduction of MSW comparative studies [38,39,40,41,42,43,44,45,46,47,48,49].

MSW LCA methodology development: With the development of LCA practices in MSWM systems, different LCA methods are also proposed based on the modification of conventional methodologies, such as an integrated LCA method encompassing both environmental and economic indicators [50], and a modular LCA framework [51].

### 1.3. Uncertainty Analysis Practice in Life Cycle Assessment Studies

Current practice suggests three types of commonly used methods for LCA uncertainty analysis, namely, sampling methods, analytical approach, and the fuzzy logic method [52]. Among various numerical methods of sampling, the Mont Carlo method is the most widely used and is performed by carrying out a large number of random samplings and propagating to generate the output probability distribution. Further, sensitivities due to the inherit uncertainties’ propagating effects are evaluated through various numerical methods such as regression coefficients [53], the Pearson correlation coefficient [54,55,56]; the Spearman correlation coefficient [57,58,59,60,61,62]; key issue analysis, [63,64,65], and the Fourier amplitude test [66].

### 1.4. Research Gaps in Municipal Solid Waste Management Life Cycle Assessments and Scope of the Present Study

The following research limitations are identified through our extensive literature review: (a) Few LCAs of MSW systems are based in China, and no existing discussions of LCA on garbage classification is especially noticed; (b) very few LCAs focus on the evaluation of effects of garbage classification on MSWM systems—on the other hand, within the life cycle of a product or service, the variations of materials and unit processes will cause different levels of energy consumption and environmental releasement, due to the variations among associated with transportations, end of life disposal and recycling scenarios, etc. [67]; (c) insufficient discussion of uncertainties decrease the reliabilities of the LCA results on MSWM systems, and uncertainty analysis is usually omitted in comparative LCA studies on MSW systems. Therefore, this study is aimed to: (a) conduct a comparative life cycle impact assessment (LCIA) study on three MSWM systems based on three garbage classification scenarios in China to investigate the environmental performances of different garbage classification scenarios; (b) carry out a comprehensive uncertainty analysis for the life cycle impacts of the studied MSWM systems, as well as the comparisons in between, to increase the knowledge on the uncertainty propagating mechanism of input data and its relationship with the output reliabilities. The results of this study are expected to provide data and information support for stakeholders, such as decision makers and the public, about the environmental benefits of garbage classification from the perspective of the life cycles of the MSWM systems. The results of LCIA and the comparative uncertainty analyses also provide information needed for trading off among different environmental impacts to improve the overall environmental performances of MSWM systems.

The main body of the paper is structured as follows: Section 2, materials and methods, will discuss garbage classification based MSWM-scenario descriptions; LCA goal and scope definition including system boundary, functional unit, and cut-off criteria; life cycle inventory data; life cycle assessment methodology and uncertainty analysis method. Section 3 will discuss the findings through an in-depth analysis of the LCIA results and uncertainty analysis. Finally, a few concluding remarks, as well as limitations of this study are briefly discussed in Section 4.

## 2. Materials and Methods

### 2.1. Garbage-Classification Based Municipal Solid Waste Management Scenarios

**Figure 1:**
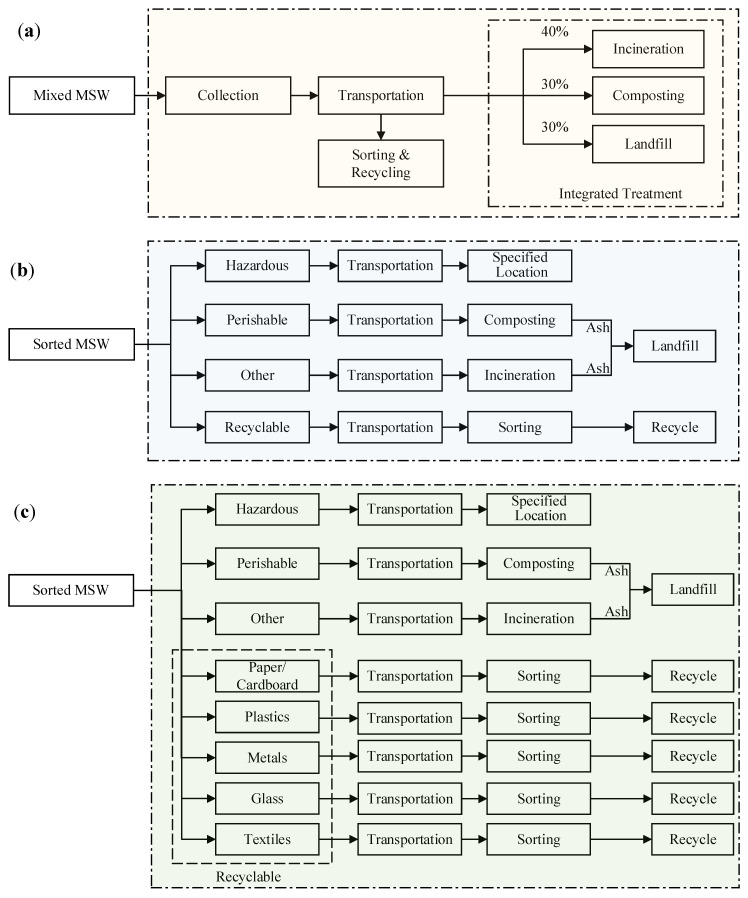
Schematic flowcharts of municipal solid waste (MSW) management systems based on proposed scenarios in China under study. (**a**) Scenario 1 (S-1); (**b**) Scenario 2 (S-2); (**c**) Scenario 3 (S-3)

Scenario 1 (S-1). This is the conventional scenario of MSWM in China and the baseline scenario for the present LCIA study. As shown in Figure 1a, under this scenario, mixed MSW is collected without sorting prior to its disposal to the collecting site, e.g., garbage carts for household garbage. The collected mixed garbage then goes through transportation and mechanical sorting, with the sorted recyclables separated and the remaining portion sent to integrated treatment facilities. The proportions of treatments in this integrated treatment systems are assumed to be 40% for incineration, 30% for composting, and 30% for landfilling [68,69].

Scenario 2 (S-2). This scenario describes an MSW management system based on an integrated treatment method incorporating four-category sorting of garbage prior to its disposal to garbage carts for collection (Figure 1b). Hazardous, perishable, recyclable, and other garbage is classified for household garbage disposal with specifically provided garbage cans available on the collecting sites. The perishable garbage is collected and transported to local landfill sites; the hazardous garbage is collected and transported to specific treatment facility; the recyclable garbage is collected and sorted to be delivered for reuse; other garbage is sent to the incineration facility. Ashes from composting and incineration are then collected and sent to landfill sites for landfilling.

Scenario 3 (S-3). This is an improved version of S-2 with enhanced efforts of classification for recyclables, under which the recyclable garbage is further classified into five categories, namely, paper/cardboard, plastics, metals, glass and textiles. The further classified and sorted garbage is disposed into specified garbage cans, collected, and transported to sorting facilities and treated for reuse (Figure 1c). Under this scenario, it is assumed that the recyclables are collected and transported through separate transportations services. Similar with under S-2, ashes from composting and incineration are collected and sent to landfill site for sanitary landfilling.

### 2.2. Main Waste Treatment Processes

**Figure 2:**
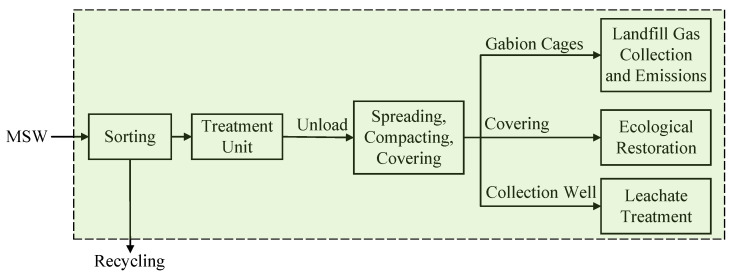
Schematic processing flowchart of municipal solid waste (MSW) sanitary landfill [70].

**Figure 3:**
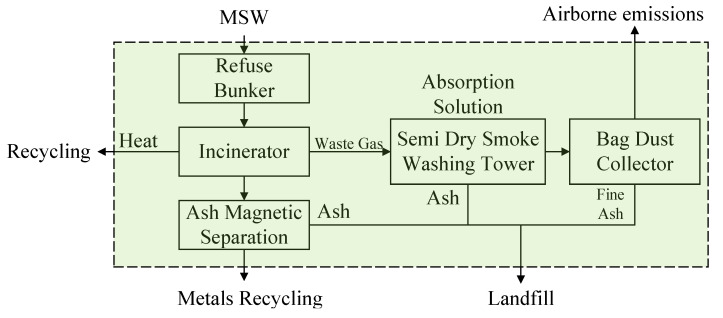
Schematic processing flowchart of municipal solid waste (MSW) incineration [68].

**Figure 4:**
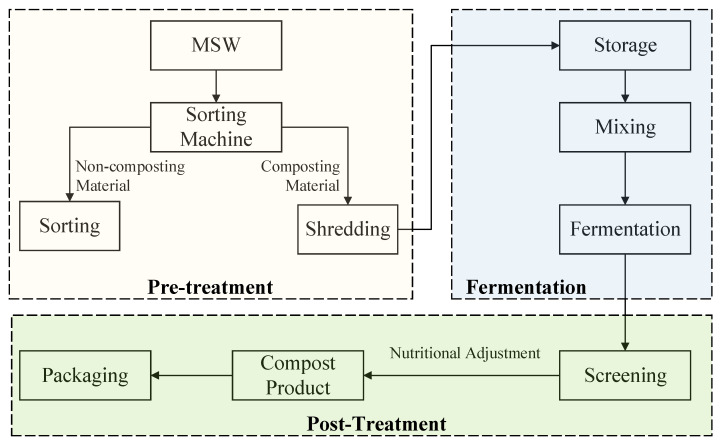
Schematic processing flowchart of municipal solid waste (MSW) composting [70].

There are three commonly used garbage treatment technologies involved in this study: sanitary landfill, incineration, and composting. The schematic processing flowcharts are shown in Figure 2, Figure 3 and Figure 4.

Landfill: Collected municipal solid garbage is treated through primary processes such as sorting, spreading, compacting, and covering, and then delivered to the landfill sites for treatment, as shown in Figure 2.

Incineration: Collected MSW is fed into the incinerator for combustion and the contained hazardous and toxicity composition is removed through incineration. The main processes involved in the incineration treatment are incinerating, dry smoke washing, dust collecting, and ash magnetic separating, as shown in Figure 3.

Composting: The three main treatment sub-processes are pretreatment, including sorting and shredding, fermentation including storage, mixing and fermentation, and post composting including screening and packaging, as shown in Figure 4.

### 2.3. Life Cycle Assessment Goal and Scope Definition

This LCA study is aimed at comparatively evaluating the environmental performances of MSWM systems based on three garbage classification scenarios that have the potentials to be applied in China. The results are expected to provide data and information support for decision makers, as well as to increase public awareness of the necessity of garbage sorting.

**Figure 5:**
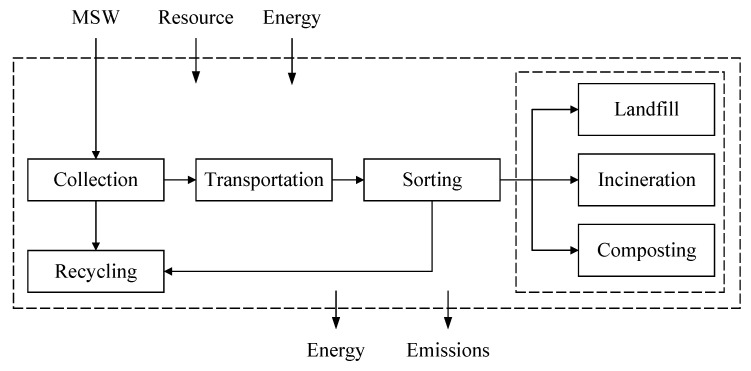
Generic system boundaries of municipal solid waste management (MSWM) systems under study. MSW, municipal solid waste.

Figure 5 shows the system boundary of the MSWM systems. Physically, the life cycle of the MSWM system encompasses the MSW collection, transportation, mechanical sorting through treatment processing such as landfill, incineration and composting, as well as all associated with consumption of materials, resources and energy.

The main function of the MSWM system is the treatment of MSW, so the functional unit of an LCA study is normally defined as the weight or volume of the solid waste [71,72]. As this case study is based on a generalized case in China, and due to the spatial-variation caused composition-proportion variations, this study defines 1 kg MSW as the functional unit for the LCA. According to current practice of LCA studies, this study uses weight and environmental significance as the cut-off criteria.

### 2.4. Life Cycle Inventory Data

Primary data, i.e., the main compositions of MSW in China, is listed in Table 1. The data is obtained from peer-reviewed publications about Chinese MSW [70,73,74,75,76]. In real world case, the compositions of MSW in different geographical locations may vary with level of economy development, living conditions, food habits and among others, however, the average local compositions have limited variations in China municipal areas. Secondary data used as the background data are taken from Ecoinvent database. As shown in Table 1, the detailed proportions of the main compositions are distributed as: paper/cardboard 13.35%; metals 1.21%, glass 3.14%, plastics 14.54%, textiles 4.45%, ceramics 3.62%, wood/bamboo 3.53%, ashes 9.07%, kitchen waste 46.54%, and hazardous 0.55%.

### 2.5. Life Cycle Impact Assessment Methodology

This study adopts IMPAC T2002+ as the LCIA method, which is a combination of Impact Assessment of Chemical Toxics (IMPACT) 2002 [77], Eco-indicator 99 [78], CML [79], and IPCC. This method provides both four damage oriented endpoint categories, namely, human health, ecosystem quality, climate change, resources, and midpoint impact categories carcinogens (kg C_2_H_3_Cl eq), non-carcinogens (kg C_2_H_3_Cl eq), respiratory inorganics (kg PM2.5 eq), ionizing radiation (Bq C−14 eq), ozone layer depletion (kg CFC−11 eq), respiratory organics (kg C_2_H_4_ eq), aquatic ecotoxicity (kg TEG water), terrestrial ecotoxicity (kg TEG soil), terrestrial acidi/nutri (kg SO_2_ eq), land occupation (m^2^org.arable), aquatic acidification (kg SO_2_ eq), aquatic eutrophication (kg PO_4_ P-lim), global warming (kg CO_2_ eq), non-renewable energy (MJ primary), and mineral extraction (MJ surplus). The normalization of IMPACT 2002+ is carried out by dividing the specific category of impact by annual average impact of an EU citizen.

### 2.6. Uncertainty Analysis

Life cycle inventory data is acquired from different sources, such as observations, peer-reviewed publications, public database, etc. Uncertainties of the collected data are unavoidably aggregated and propagated through the stepwise life cycle assessment procedures, namely, data calculation, modelling, cutting off, allocating, and computer data processing with large amount of rounding offs. Therefore, as a result, both the life cycle inventory analysis and the impact assessment results will inherit the uncertainties initiated from various data sources. Thus, a proper uncertainty analysis is crucial to validate the LCA results and ensure the reliability of the interpreted information. As triangle distribution is an effective method to determine the distribution of variables when the sample data of uncertain parameters are finite [80], for this LCA study, the uncertainties of the collected input data are assumed to be following triangular distributions, with ±0.05% errors suggested. Monte Carlo has been widely applied for the uncertainty analysis in many LCA studies [81,82]. Monte Carlo uncertainty analysis is normally carried out through the following steps: (a) definition of the probability distributions of input data; (b) definition of the output values as numerical functions of inputs; (c) carrying out random sampling for input data for a large number of runs; (d) generation of probability distribution of outputs. The stepwise procedures can be expressed as below:

(1) $$y=y\left(x_{1},x_{2},\ldots x_{M}\right),$$

(2) $$y_{i}=y\left(x_{1i},x_{2i},\ldots x_{Mi}\right),$$

(3) $$\bar{y}=\frac{1}{N}\sum_{i=1}^{N}y_{i},$$

(4) $$\sigma^{2}=\frac{1}{N-1}\overset{N}{\underset{i=1}{\sum }}{\left(y_{i}-\bar{y}\right)}^{2},$$

where, $\left(x_{1i},x_{2i},\ldots x_{Mi}\right)$, *i*th random sampling of input data *x*_1_, *x*_2_, *… x_M_*; y, an arbitrary output value, as a function of input parameters *x*_1_, *x*_2_, *… x_M_*; $y_{i}$, the output parameter value decided by the *i*th random sampling; $\bar{y}$, mean output of parameter y after N random sampling; σ, standard deviation of output parameter y after N random sampling.

## 3. Results and Discussion

### 3.1. Life Cycle Impact Assessment

#### 3.1.1. Midpoint Impacts

**Figure 6:**
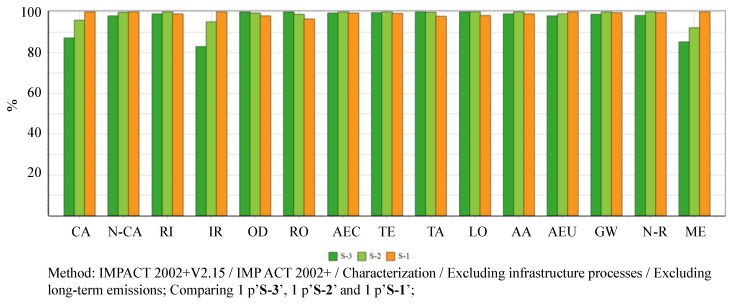
Midpoint life cycle impacts of studied municipal solid waste management (MSWM)systems based on Scenarios S1–3. Notes: CA, climate change; N-CA, non-carcinogens; RI, respiratory inorganics; IR, ionizing radiation; OD, ozone layer depletion; RO, respiratory organics; AEC, aquatic ecotoxicity; TE, terrestrial ecotoxicity; TA, terrestrial acid/nutri; LO, land occupation; AA, aquatic acidification; AEU, aquatic eutrophication; GW, global warming; N-R, non-renewable energy; and, ME, mineral extraction; IMPACT, Impact Assessment of Chemical Toxics; S, scenario.

The midpoint life cycle impacts are calculated through IMPACT 2002+ method, with the comparisons of all categories shown in Figure 6 and detailed numerical valued available in Table S1. The calculated results of the midpoint impacts show that: (a) S-1 has the most significant impacts in categories: carcinogens 2.771E−01 kg C_2_H_3_Cl, non-carcinogens 4.199E−01 kg C_2_H_3_Cl eq, ionizing radiation 6.300E+01 Bq C−14 eq, aquatic eutrophication 3.768E−03 kg PO_4_ P-lim, mineral extraction 6.016E−01 MJ surplus, and the least significant impacts in categories: aquatic ecotoxicity 2.733E+03 kg TEG water, terrestrial ecotoxicity 9.386E+02 kg TEG soil, terrestrial acid/nutri 3.731E−01 kg SO_2_ eq, and land occupation 1.047E+01 m^2^org.arable; (b) S-2 has the highest impacts in categories: respiratory inorganics 2.159E−02 kg PM2.5 eq, aquatic ecotoxicity 2.749E+03 kg TEG water, terrestrial ecotoxicity 9.464E+02 kg TEG soil, aquatic eutrophication 7.279E−02 kg SO_2_ eq, global warming 1.109E+01 kg CO_2_ eq, non-renewable energy 1.880E+02 MJ primary; (c) S-3 is the most significant contributor in such impact categories as ozone layer depletion 2.277E−06 kg CFC−11 eq, respiratory organics 1.214E−02 kg C_2_H_4_ eq, terrestrial acid/nutri 3.811E−01 kg SO_2_ eq, land occupation 1.065E+01 m^2^org.arable, as well as contributing the least significant impacts in categories: carcinogens 2.416E−01 kg C_2_H_3_Cl eq, non-carcinogens 4.118E−01 kg C_2_H_3_Cl eq, respiratory inorganics 2.137E−02 kg PM2.5 eq, ionizing radiation 5.225E+01 Bq C−14 eq, aquatic acidification 7.203E−02 kg SO_2_ eq, aquatic eutrophication 3.693E−03 kg PO_4_ P-lim, global warming 1.097E+01 kg CO_2_ eq, and mineral extraction 5.131E−01 MJ surplus.

#### 3.1.2. Endpoint Impacts

**Figure 7:**
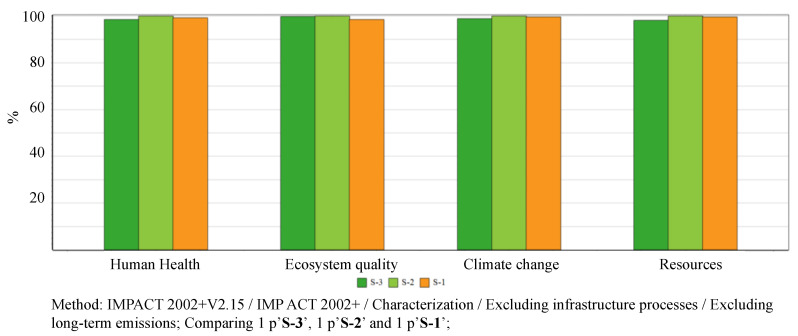
Endpoint life cycle impacts, i.e., damages via municipal solid waste management (MSWM)systems based on S1–3.

IMPACT 2002+ method calculates the damage of human health per contributions from midpoint impact categories: carcinogens, non-carcinogens, respiratory inorganics, ionizing radiation, ozone depletion, respiratory organics and; damage of ecosystem quality per: aquatic ecotoxicity, terrestrial ecotoxicity, terrestrial acid/nutri, land occupation, aquatic acidification, and aquatic eutrophication; damage of climate change per: midpoint impact category global warming; and damage of resources per non-renewable energy and mineral extraction. The comparative endpoint life cycle impacts, i.e., damages are calculated and shown in Figure 7. The main finding of the endpoint LCIA results demonstrate that: (a) S-1 is the least significant contributor of ecosystem quality, with an amount of 1.9357E+01 PDF*m^2^*yr; (b) S-2 is the most significant contributor of human health with an amount of 1.7070E−05 DALY, ecosystem quality with an amount of 1.9631E+01 PDF*m^2^*yr, climate change 1.1093E+01 kg CO_2_ eq, resources with amount of 1.8856E+02 MJ primary; (c) S-3 contributes the least significant to damages of human health with 1.6827E−05 DALY, climate change with 1.0965E+01 kg CO_2_ eq, and resources depletion with 1.8508E+02 MJ primary. The detailed contributions per midpoint impact categories of the three scenarios are shown in Table 2.

#### 3.1.3. Single Scored Impacts

**Figure 8:**
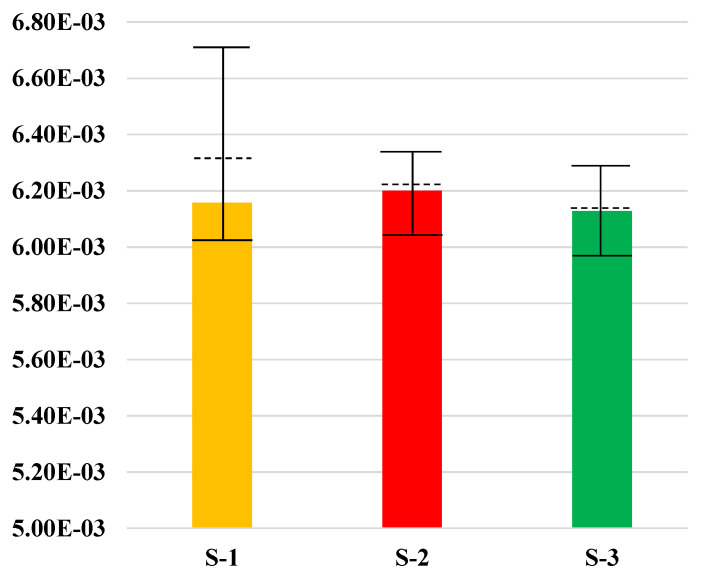
Single scored life cycle impacts with 95.0% confidence intervals calculated by Monte Carlo uncertainty analysis based on S1–3. Means, 2.5 to 97.5% confidence intervals are: S-1, 6.3140E−03, 6.0029E−3~6.6557E−03; S-2, 6.2086E−03, 6.0506E−03~6.3681E−03; S-3, 6.1342E−03, and 5.9790E−03~6.2883E−03. (See Tables S2 and S3 for details).

IMPACT 2002+ provides such a way that the values of different impact categories are grouped, weighted and added up to be a single score index. As the levels of life cycle impacts that we intend to interpret are very likely dependent on the actual audience, a single scored impact gives the most straightforward understandable approach to compare the environmental performance of a product system with others. With all the weighted sores of midpoint impact categories added together, the single scored life cycle impacts of S-1, S-2, and S-3 are 6.16, 6.2, and 6.13 mPt. The most dominant midpoint impacts for the scores are respiratory inorganics, global warming and non-renewable energy. Their weighted scores are 2.11, 2.13, and 2.11 mPt; 1.12, 1.12, and 1.11 mPt; 1.23, 1.24, and 1.21 mPt, for S-1, S-2, and S-3 respectively, as shown in Figure 8.

### 3.2. Uncertainty Analysis

**Figure 9:**
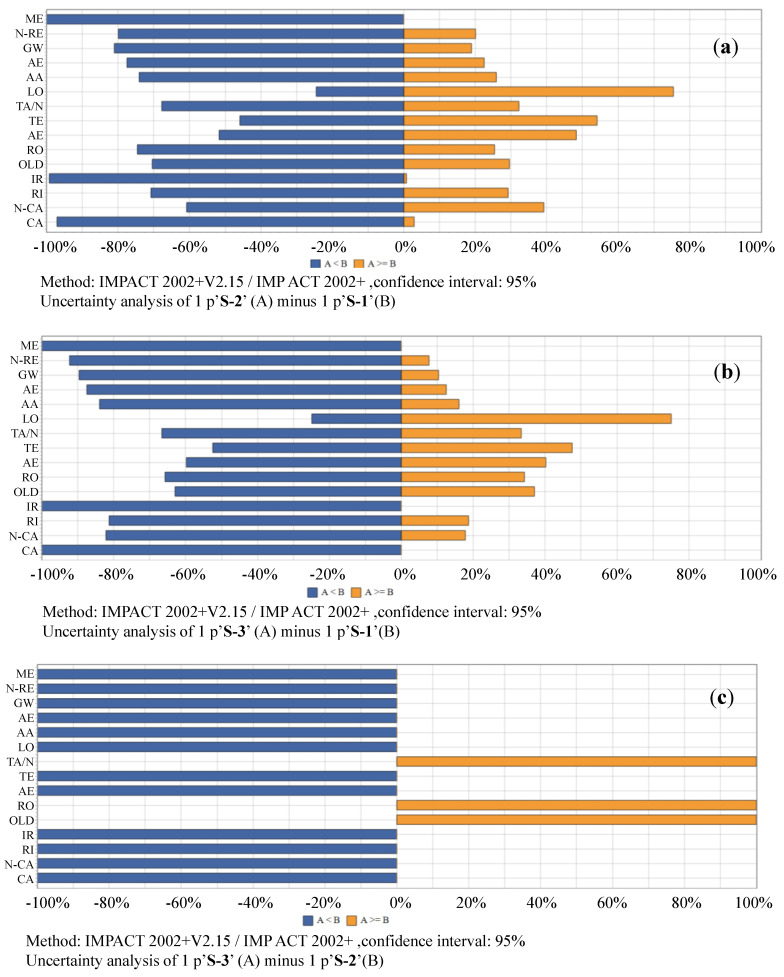
Probability distributions of midpoint life cycle impact comparisons in between scenarios. (**a**) comparison between S-1 vs. S-2, (**b**) comparison between S-1 vs. S-3, (**c**) comparison between S-2 vs. S-3. Notes: with confidence interval of 95%, simulated by Monte Carlo sampling, 10,000 runs. CA, climate change; N-CA, non-carcinogens; RI, respiratory inorganics; IR, ionizing radiation; OD, ozone layer depletion; RO, respiratory organics; AEC, aquatic ecotoxicity; TE, terrestrial ecotoxicity; TA, terrestrial acid/nutri; LO, land occupation; AA, aquatic acidification; AEU, aquatic eutrophication; GW, global warming; N-R, non-renewable energy; and, ME, mineral extraction; IMPACT, Impact Assessment of Chemical Toxics; S, scenario.

**Figure 10:**
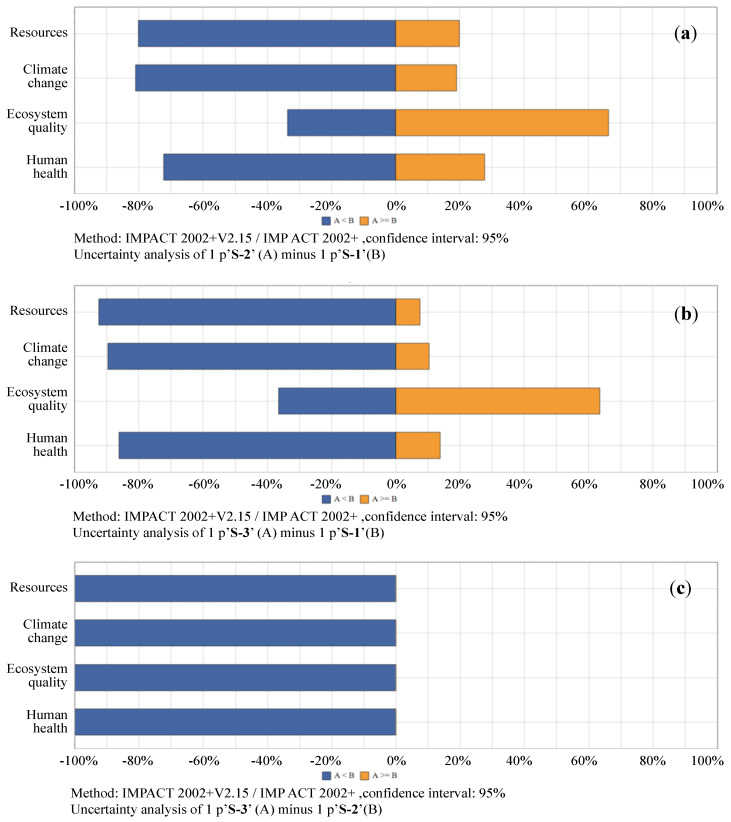
Probability distributions of damages, i.e., endpoint life cycle impact comparisons in between scenarios. (**a**) comparison between S-1 vs. S-2, (**b**) comparison between S-1 vs. S-3, (**c**) comparison between S-2 vs. S-3. Notes: with confidence interval of 95%, simulated by Monte Carlo sampling, 10,000 runs; IMPACT, Impact Assessment of Chemical Toxics; S, scenario.

**Figure 11:**
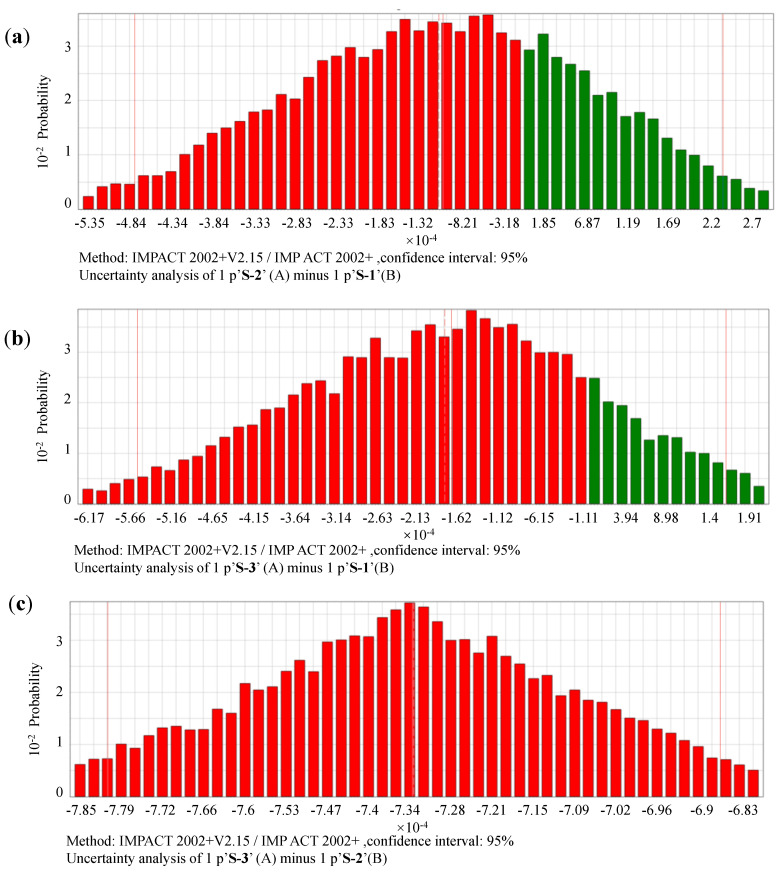
Probability distributions of single scored life cycle impacts in between scenarios. (**a**) comparison between S-1 vs. S-2, (**b**) comparison between S-1 vs. S-3, (**c**) comparison between S-2 vs. S-3. Notes: with confidence interval of 95%, simulated by Monte Carlo sampling, 10,000 runs; IMPACT, Impact Assessment of Chemical Toxics; S, scenario.

With application of Monte Carlo method, 10,000 run samplings are conducted to test the probability distributions of the differences for the same impact categories in between different scenarios. The probabilities of the subtractions of impacts in between S-1 vs. S-2, S-1 vs. S-3, as well as S-2 vs. S-3. The testing results for midpoint, damage, i.e., endpoint, and single scored impacts are shown as Figure 9, Figure 10 and Figure 11. The associated with details of their numerical values are listed in Tables S2 and S3.

#### 3.2.1. Uncertainties of Midpoint Impact Comparisons in Between Scenario 1, Scenario 2 and Scenario 3

S-1 vs. S-2:aAs shown in Figure 9a and Table S4, the uncertainty analyses show that with a confidence interval of 95%, the mean values of the differences (S-2’s minus S-1’s) for each of the midpoint life cycle impacts are expected to be −1.730E−03 kg SO_2_ eq for aquatic acidification, −2.798E+00 kg TEG water for aquatic ecotoxicity, −7.195E−05 kg PO_4_ P-lim for aquatic eutrophication, −1.251E−02 kg C_2_H_3_Cl eq for carcinogens, −3.734E−01 kg CO_2_ eq for global warming, −8.306E+00 BqC−14 eq for ionizing radiation, 1.822E−01 m2org.arable for land occupation, −4.883E−02 MJ surplus for mineral extraction, −2.866E−03 kg C_2_H_3_Cl eq for non-carcinogens, −5.770E+00 MJ primary for non-renewable energy, −4.712E−08 kg CFC−11 eq for ozone layer depletion, −4.134E−04 kg PM2.5 eq for respiratory inorganics, −4.917E−04 kg C_2_H_4_ eq for respiratory organics, and −7.792E−03 kg SO_2_ eq for terrestrial acid/nutri, 2.443E+00 kg TEG soil for terrestrial ecotoxicity. The probabilities that S-1 ≥ S-2 for the midpoint impacts are: 74.1% for aquatic acidification, 51.71% for aquatic ecotoxicity, 77.45% for aquatic eutrophication, 97.06% for carcinogens, 81.06% for global warming, 99.18% for ionizing radiation, 24.57% for land occupation, 99.98% for mineral extraction, 60.78% for non-carcinogens, 79.88% for non-renewable energy, 70.46% for ozone layer depletion, 70.82% for respiratory inorganics, 74.53% for respiratory organics, 67.81% for terrestrial acid/nutri, and 45.93% for terrestrial ecotoxicity. Land occupation and terrestrial ecotoxicity are the only two impact categories where the probabilities of S-2 being greater than S-1 are over 50%. This indicates that it is more likely that S-2 has a more friendly environmental performance than S-1 in most of the midpoint impacts excluding land occupation and terrestrial ecotoxicity.

S-1 vs. S-3: as shown in Figure 9b and Table S5, with a confidence interval of 95%, the mean values of the differences (S-3’s minus S-1’s) for each of the midpoint life cycle impacts are expected to be −2.457E−03 kg SO_2_ eq for aquatic acidification, −1.762E+01 kg TEG water for aquatic ecotoxicity, −1.100E−04 kg PO_4_ P-lim for aquatic eutrophication, −3.668E−02 kg C_2_H_3_Cl eq for carcinogens, −4.942E−01 kg CO_2_ eq for global warming, −2.169E+01 BqC−14 eq for ionizing radiation, 1.786E−01 m^2^org.arable for land occupation, −9.020E−02 MJ surplus for mineral extraction, −9.892E−03 kg C_2_H_3_Cl eq for non-carcinogens, −9.097E+00 MJ primary for non-renewable energy, −3.130E−08 kg CFC−11 eq for ozone layer depletion, −6.218E−04 kg PM2.5 eq for respiratory inorganics, −3.400E−04 kg C_2_H_4_ eq for respiratory organics, −7.170E−03 kg SO_2_ eq for terrestrial acid/nutri, and −1.574E+00 kg TEG soil for terrestrial ecotoxicity. The probabilities that S-3 ≥ S-1 for the midpoint impacts are: 16.05% for aquatic acidification, 40.25% for aquatic ecotoxicity, 12.55% for aquatic eutrophication, 0% for carcinogens, 10.35% for global warming, 0% for ionizing radiation, 75.06% for land occupation, 0% for mineral extraction, 17.82% for non-carcinogens, 7.67% for non-renewable energy, 36.98% for ozone layer depletion, 18.67% for respiratory inorganics, 34.29% for respiratory organics, 33.33% for terrestrial acid/nutri, and 47.54% for terrestrial ecotoxicity. Land occupation is the only impact category where the probability of S-3 being greater than S-1 are over 50%. This indicates that it is likely that S-3 has more friendly environmental performance than S-1 in most of the midpoint impacts excluding land occupation.

S-2 vs. S-3: as shown in Figure 9c and Table S6, with a confidence interval of 95%, the mean values of the differences (S-3’s minus S-2’s) for each of the midpoint life cycle impacts are expected to be −7.693E−04 kg SO_2_ eq for aquatic acidification, −1.564E+01 kg TEG water for aquatic ecotoxicity, −3.914E−05 kg PO_4_ P-lim for aquatic eutrophication, −2.424E−02 kg C_2_H_3_Cl eq for carcinogens, −1.278E−01 kg CO_2_ eq for global warming, −1.345E+01 Bq C−14 eq for ionizing radiation, −6.180E−03 m^2^org.arable for land occupation, −4.150E−02 MJ surplus for mineral extraction, −7.134E−03 kg C_2_H_3_Cl eq for non-carcinogens, −3.442E+00 MJ primary for non-renewable energy, 1.449E−08 kg CFC−11 eq for ozone layer depletion, −2.205E−04 kg PM2.5 eq for respiratory inorganics, 1.400E−04 kg C_2_H_4_ eq for respiratory organics, 3.763E−04 kg SO_2_ eq for terrestrial acid/nutri, and −4.293E+00 kg TEG soil for terrestrial ecotoxicity. The probabilities that S-3 ≥ S-1 for the midpoint impacts are: 0% for aquatic acidification, aquatic ecotoxicity, aquatic eutrophication, carcinogens, global warming, ionizing radiation, land occupation, mineral extraction, non-carcinogens, non-renewable energy, respiratory inorganics, terrestrial ecotoxicity; 100% for ozone layer depletion, respiratory organics, and terrestrial acid/nutri. This indicates that S-3 has better environmental performance than S-2 in all midpoint categories except for land occupation, respiratory organics, and terrestrial acid/nutri.

#### 3.2.2. Uncertainties of Damages, i.e., Endpoint Impact Comparisons in Between Scenario 1, Scenario 2 and Scenario 3

S-1 vs. S-2: as illustrated in Figure 10a, and with detailed numerical values available in Table S7, the results of Monte Carlo sampling with 10,000 runs show that with a confidence interval of 95%, the mean values of the differences (S-2’s minus S-1’s) for each of the endpoint life cycle impacts are expected to be −3.73E−01 kg CO_2_ eq for climate change, 2.10E−01 PDF*m^2^*yr for ecosystem quality, −3.35E−07 DALY for human health, −5.82E+00 MJ primary for resources. The probabilities that S-2 ≥ S-1 for the endpoint impacts are: 18.94% for climate change, 66.3% for ecosystem quality, 27.74% for human health, and 19.91% for resources.

S-1 vs. S-3: as shown in Figure 10b and Table S8, the means of the differences (S-3’s minus S-1’s) for each of the endpoint life cycle impacts are calculated to be −4.94E−01 kg CO_2_ eq for climate change, 1.74E−01 PDF*m^2^*yr for ecosystem quality, −5.71E−07 DALY for human health, −9.19E+00 MJ primary for resources. The probabilities that S-3 ≥ S-1 for the endpoint impacts are: 10.35% for climate change, 63.51% for ecosystem quality, 13.77% for human health, and 7.49% for resources.

S-2 vs. S-3: as shown in Figure 10c and Table S9, the means of the differences (S-3’s minus S-2’s) for each of the endpoint life cycle impacts are −1.28E−01 kg CO_2_ eq for climate change, −4.11E−02 PDF*m^2^*yr for ecosystem quality, −2.45E−07 DALY for human health, −3.48E+00 MJ primary for resources. All the probabilities that S-3 ≥ S-2 for the endpoint impacts are calculated to be 0%.

#### 3.2.3. Uncertainties of Single-Scored Impacts in Between Scenario 1, Scenario 2, and Scenario 3

As illustrated in Tables S10–S12, with a confidence interval of 95%, the mean values of the single score impact differences in between S-1, S-2, and S-3 are −1.08E−04 for S-2 minus S-1, −1.78E−04 for S-3 minus S-1, and −1.78E−04 for S-3 minus S-2. The probability distribution of the differences in between single scores of S-1 vs. S-2, S-1 vs. S-3, S-2 vs. S-3 are shown in Figure 11a–c.

The probabilities that S-2 ≥ S-1, S-3 ≥ S-1, and S-3 ≥ S-2, are 29.43%, 17.15%, and 0%. This indicates that it is more likely that S-3 has the best environmental performance, followed by S-1 and then S-2. The reason that S-2 has a larger score than S-1 may be caused by the consumption of energy and resources, as its treatment of the sorted garbage involves more transportation and sorting processing. However, with a more detailed sorting requirements, S-3 shows better environmental performance than S-2 in terms of a single score index.

## 4. Conclusions

A comparative LCIA study was performed for MSWM systems based on three garbage classification scenarios in China. The LCIA results indicate that systems based on various scenarios show significant variations for the studied environmental impacts. For midpoint impacts, S-1 is the most significant contributor to categories: carcinogens, non-carcinogens, ionizing radiation, aquatic eutrophication, mineral extraction, and the least significant contributors in categories aquatic ecotoxicity, terrestrial ecotoxicity, terrestrial acid/nutri, and land occupation; S-2 has the highest impacts in categories: respiratory inorganics, aquatic ecotoxicity, terrestrial ecotoxicity, aquatic eutrophication, global warming, non-renewable energy; S-3 is the most significant contributor to ozone layer depletion, respiratory organics, terrestrial acid/nutri, land occupation, and contributes the least significant impacts in carcinogens, non-carcinogens, respiratory inorganics, ionizing radiation, aquatic acidification, aquatic eutrophication, global warming, and mineral extraction.

As for damage assessment results, S-1 is the least significant contributor of ecosystem quality; S-2 is the most significant contributor of human health; ecosystem quality, and climate change; S-3 contributes the least significant to damages of human health, climate change, and resources depletion. Finally, S-2 is calculated to be the most significant environmental impact contributor, followed by S-1 and S-3, from the perspective of single scoring the weighted midpoint impacts’ overall environmental effects.

Monte Carlo uncertainty analysis was conducted to quantitatively investigate the probabilistic nature of the comparisons in between the environmental impacts of the three proposed scenarios. The probabilities of the comparisons of each life cycle impact category for midpoint, endpoint, and single score for every two scenarios are calculated and discussed. The probability distributions of the comparisons show that different garbage classification scenarios have their own environmental-friendly superiority is various of impact categories.

As per the results of LCIA and Monte Carlo uncertainty analysis, it is concluded that: (1) the environmental effects of garbage classification are likely to be positive, by comparison with MSWM systems without classification; (2) more detailed-classification based MSWM system has better environmental performance; (3) tradeoffs should be carefully made for real world practice of MSWM system designing; (4) the transportation services, garbage treatment facilities and processing techniques must be updated and optimized for better implementation of garbage classification laws and regulations; (5) it is necessary to increase the public awareness of the benefits of garbage classification from social, economic, environmental and psychological perspectives. Due to availability of data, the spacial and temporal variations are not considered, but should however be included in our future study. 

## Figures and Tables

**Table 1:** Compositions of mixed municipal solid waste (MSW) in China [70,73,74,75,76].

Composition	Proportion (%)	Detailed Composition
Paper/cardboard	13.35	Waste books, newspapers, paper boxes, waste papers, waste toilet papers, sanitary napkins etc.
Metals	1.21	Aluminum cans, tin cans, wasted metal components and parts (excluding button batteries), etc.
Glass	3.14	Glass bottles, bowls, containers, handicraft, etc.
Plastics	14.54	Plastic bottles, packages, wet contaminated plastic, rubbers, leatherware, etc.
Textiles	4.45	Wasted cloth, cotton textiles, etc.
Ceramics	3.62	Wasted bricks, tiles, ceramics, stones, cement, etc.
Wood/bamboo	3.53	Wasted wood, bamboo, flowers, plants, etc.
Ashes	9.07	Dirt, ash, lime sands, etc.
Kitchen waste	46.54	Wasted plant foods, meats, fruits, etc.
Hazardous	0.55	Wasted batteries, paints, pesticides, etc.

**Table 2:** Damage assessment results, i.e., endpoint life cycle impacts of studied municipal solid waste (MSW)systems based on S1–3.

Damage Category	Impact Category	Unit	S-1	S-2	S-3
Human health	Human health total	DALY	1.6959E−05	1.7070E−05	1.6827E−05
	Carcinogens	DALY	7.763E−07	7.445E−07	6.766E−07
	Non-carcinogens	DALY	1.179E−06	1.176E−06	1.156E−06
	Respiratory inorganics	DALY	1.500E−05	1.515E−05	1.499E−05
	Ionizing radiation	DALY	2.677E−08	2.562E−08	2.279E−08
	Ozone layer depletion	DALY	2.346E−09	2.377E−09	2.392E−09
	Respiratory organics	DALY	2.494E−08	2.556E−08	2.586E−08
Ecosystem quality	Ecosystem quality total	PDF*m^2^*yr	1.9357E+01	1.9631E+01	1.9591E+01
	Aquatic ecotoxicity	PDF*m^2^*yr	1.376E−01	1.384E−01	1.376E−01
	Terrestrial ecotoxicity	PDF*m^2^*yr	7.445E+00	7.506E+00	7.472E+00
	Terrestrial acid/nutri	PDF*m^2^*yr	3.880E−01	3.959E−01	3.963E−01
	Land occupation	PDF*m^2^*yr	1.141E+01	1.161E+01	1.160E+01
	Aquatic acidification	-	-	-	-
	Aquatic eutrophication	-	-	-	-
Climate change	Climate change total	kg CO_2_ eq	1.1052E+01	1.1093E+01	1.0965E+01
	Global warming	kg CO_2_ eq	1.105E+01	1.109E+01	1.097E+01
Resources	Resources total	MJ primary	1.8796E+02	1.8856E+02	1.8508E+02
	Non-renewable energy	MJ primary	1.874E+02	1.880E+02	1.846E+02
	Mineral extraction	MJ primary	6.016E−01	5.546E−01	5.131E−01

DALY is the acronym of Disability-Adjusted Life Year, quantifying the burden of disease from mortality and morbidity [83]; PDF*m^2^*yr, potentially disappeared fraction of species * square meters * year; kg CO_2_ eq, kilogram carbon dioxide equivalents; MJ, megajoule.

## Supplementary Materials

The following is available online at https://www.mdpi.com/1660-4601/17/15/5310/s1, Table S1: Comparative Midpoint Life Cycle Impact Assessment Results, Table S2: Comparative Single Scored Life Cycle Impact Assessment Results, Table S3: Uncertainty analysis results for single scored life cycle impact assessment, Table S4: Uncertainty analysis results for comparison between S-1 and S-2, midpoint,1 p ‘S-2’ (A) minus 1 p ‘S-1’ (B), Table S5: Uncertainty analysis results for comparison between S-1 and S-3, midpoint,1 p ‘S-3’ (A) minus 1 p ‘S-1’ (B), Table S6: Uncertainty analysis results for comparison between S-2 and S-3, midpoint,1 p ‘S-3’ (A) minus 1 p ‘S-2’ (B), Table S7: Uncertainty analysis results for comparison between S-1 and S-2, endpoint,1 p ‘S-2’ (A) minus 1 p ‘S-1’ (B), Table S8: Uncertainty analysis results for comparison between S-1 and S-3, endpoint,1 p ‘S-3’ (A) minus 1 p ‘S-1’ (B), Table S9: Uncertainty analysis results for comparison between S-2 and S-3, endpoint,1 p ‘S-3’ (A) minus 1 p ‘S-2’ (B), Table S10: Uncertainty analysis results for comparison between S-1 and S-2, Single score,1 p ‘S-2’ (A) minus 1 p ‘S-1’ (B), Table S11: Uncertainty analysis results for comparison between S-1 and S-3, Single score,1 p ‘S-3’ (A) minus 1 p ‘S-1’ (B), Table S12: Uncertainty analysis results for comparison between S-2 and S-3, Single score,1 p ‘S-3’ (A) minus 1 p ‘S-2’ (B).
