# Supplementary material for: Life Cycle Impact Assessment of Garbage-Classification Based Municipal Solid Waste Management Systems: A Comparative Case Study in China
Source: Int J Environ Res Public Health. 2020 Jul 23;17(15):5310. doi: 10.3390/ijerph17155310 (PMC7432649; doi:10.3390/ijerph17155310)
Supplement: Supplementary file 1 [file ijerph-17-05310-s001.pdf]

Supplementary Materials

Table S1

Comparative Midpoint Life Cycle Impact Assessment Results

Impact category	Unit	S-1	S-2	S-3
Carcinogens	kg C2H3Cl eq	2.771E-01	2.658E-01	2.416E-01
Non-carcinogens	kg C2H3Cl eq	4.199E-01	4.188E-01	4.118E-01
Respiratory inorganics	kg PM2.5 eq	2.138E-02	2.159E-02	2.137E-02
Ionizing radiation	Bq C-14 eq	6.300E+01	5.994E+01	5.225E+01
Ozone layer depletion	kg CFC-11 eq	2.233E-06	2.263E-06	2.277E-06
Respiratory organics	kg C2H4 eq	1.171E-02	1.200E-02	1.214E-02
Aquatic ecotoxicity	kg TEG water	2.733E+03	2.749E+03	2.734E+03
Terrestrial ecotoxicity	kg TEG soil	9.386E+02	9.464E+02	9.424E+02
Terrestrial acid/nutri	kg SO2 eq	3.731E-01	3.807E-01	3.811E-01
Land occupation	m2org.arable	1.047E+01	1.065E+01	1.065E+01
Aquatic acidification	kg SO2 eq	7.207E-02	7.279E-02	7.203E-02
Aquatic eutrophication	kg PO4 P-lim	3.768E-03	3.732E-03	3.693E-03
Global warming	kg CO2 eq	1.105E+01	1.109E+01	1.097E+01
Non-renewable energy	MJ primary	1.874E+02	1.880E+02	1.846E+02
Mineral extraction	MJ surplus	6.016E-01	5.546E-01	5.131E-01

Table S2

Comparative Single Scored Life Cycle Impact Assessment Results

Impact category	Unit	S-1	S-2	S-3
Total	mPt	6.157E+00	6.201E+00	6.128E+00
Carcinogens	mPt	1.094E-01	1.049E-01	9.537E-02
Non-carcinogens	mPt	1.658E-01	1.654E-01	1.626E-01
Respiratory inorganics	mPt	2.110E+00	2.131E+00	2.109E+00
Ionizing radiation	mPt	1.865E-03	1.775E-03	1.547E-03
Ozone layer depletion	mPt	3.306E-04	3.350E-04	3.371E-04
Respiratory organics	mPt	3.516E-03	3.604E-03	3.646E-03
Aquatic ecotoxicity	mPt	1.002E-02	1.007E-02	1.002E-02
Terrestrial ecotoxicity	mPt	5.420E-01	5.465E-01	5.441E-01
Terrestrial acid/nutri	mPt	2.833E-02	2.890E-02	2.893E-02
Land occupation	mPt	8.328E-01	8.476E-01	8.471E-01
Aquatic acidification	mPt	0.000E+00	0.000E+00	0.000E+00
Aquatic eutrophication	mPt	0.000E+00	0.000E+00	0.000E+00
Global warming	mPt	1.116E+00	1.120E+00	1.108E+00
Non-renewable energy	mPt	1.233E+00	1.237E+00	1.214E+00
Mineral extraction	mPt	3.958E-03	3.649E-03	3.376E-03

Table S3

Uncertainty analysis results for single scored life cycle impact assessment

Scenarios	Mean (Pt)	Median (Pt)	SD (Pt)	CV (Pt)	2.5% (Pt)	97.5% (Pt)	SEM (Pt)
S-1	6.3140E-03	6.3080E-03	1.6772E-04	2.6563E+00	6.0029E-03	6.6557E-03	1.6772E-06
S-2	6.2086E-03	6.2087E-03	8.0573E-05	1.2978E+00	6.0506E-03	6.3681E-03	8.0573E-07
S-3	6.1342E-03	6.1334E-03	7.9203E-05	1.2912E+00	5.9790E-03	6.2883E-03	7.9203E-07

Table S4

Uncertainty analysis results for comparison between S-1 and S-2, midpoint,1 p 'S-2' (A) minus 1 p 'S-1' (B).

Impact category	A >= B (%)	Mean	Median	SD	CV	2.5%	97.5%	SEM
Aquatic acidification	25.9	-1.73E-03	-1.60E-03	2.47E-03	-1.43E+02	-6.72E-03	2.71E-03	2.47E-05
Aquatic ecotoxicity	48.29	-2.80E+00	-3.01E+00	6.70E+01	-2.39E+03	-1.32E+02	1.26E+02	6.70E-01
Aquatic eutrophication	22.55	-7.19E-05	-7.20E-05	9.14E-05	-1.27E+02	-2.50E-04	1.03E-04	9.14E-07
Carcinogens	2.94	-1.25E-02	-1.25E-02	6.71E-03	-5.36E+01	-2.55E-02	3.70E-04	6.71E-05
Global warming	18.94	-3.73E-01	-3.45E-01	4.05E-01	-1.08E+02	-1.20E+00	3.45E-01	4.05E-03
Ionizing radiation	0.82	-8.31E+00	-8.23E+00	3.76E+00	-4.53E+01	-1.57E+01	-1.25E+00	3.76E-02
Land occupation	75.43	1.82E-01	1.81E-01	2.61E-01	1.43E+02	-3.25E-01	6.85E-01	2.61E-03
Mineral extraction	0.02	-4.88E-02	-4.88E-02	1.45E-02	-2.97E+01	-7.70E-02	-2.09E-02	1.45E-04
Non-carcinogens	39.22	-2.87E-03	-2.93E-03	1.01E-02	-3.52E+02	-2.23E-02	1.66E-02	1.01E-04
Non-renewable energy	20.12	-5.77E+00	-5.41E+00	6.58E+00	-1.14E+02	-1.91E+01	6.06E+00	6.58E-02
Ozone layer depletion	29.54	-4.71E-08	-4.30E-08	7.98E-08	-1.69E+02	-2.09E-07	9.66E-08	7.98E-10
Respiratory inorganics	29.18	-4.13E-04	-3.90E-04	6.91E-04	-1.67E+02	-1.80E-03	8.53E-04	6.91E-06
Respiratory organics	25.47	-4.92E-04	-4.14E-04	6.48E-04	-1.32E+02	-1.84E-03	5.63E-04	6.48E-06
Terrestrial acid/nutri	32.19	-7.79E-03	-6.69E-03	1.44E-02	-1.84E+02	-3.73E-02	1.72E-02	1.44E-04
Terrestrial ecotoxicity	54.07	2.44E+00	2.38E+00	2.30E+01	9.41E+02	-4.19E+01	4.68E+01	2.30E-01
Confidence interval:	95%							

Table S5

Uncertainty analysis results for comparison between S-1 and S-3, midpoint,1 p 'S-3' (A) minus 1 p 'S-1' (B).

Impact category	A >= B(%)	Mean	Median	SD	CV	2.5%	97.5%	SEM
Aquatic acidification	16.05	-2.5E-03	-2.3E-03	2.5E-03	-1.0E+02	-7.6E-03	2.0E-03	2.5E-05
Aquatic ecotoxicity	40.25	-1.8E+01	-1.7E+01	6.8E+01	-3.9E+02	-1.5E+02	1.1E+02	6.8E-01
Aquatic eutrophication	12.55	-1.1E-04	-1.1E-04	9.3E-05	-8.5E+01	-2.9E-04	6.6E-05	9.3E-07
Carcinogens	0	-3.7E-02	-3.7E-02	6.6E-03	-1.8E+01	-5.0E-02	-2.4E-02	6.6E-05
Global warming	10.35	-4.9E-01	-4.6E-01	4.0E-01	-8.2E+01	-1.3E+00	2.3E-01	4.0E-03
Ionizing radiation	0	-2.2E+01	-2.2E+01	3.7E+00	-1.7E+01	-2.9E+01	-1.5E+01	3.7E-02
Land occupation	75.06	1.8E-01	1.8E-01	2.6E-01	1.5E+02	-3.4E-01	6.8E-01	2.6E-03
Mineral extraction	0	-9.0E-02	-9.0E-02	1.4E-02	-1.6E+01	-1.2E-01	-6.3E-02	1.4E-04
Non-carcinogens	17.82	-9.9E-03	-9.9E-03	1.0E-02	-1.0E+02	-3.0E-02	9.5E-03	1.0E-04
Non-renewable energy	7.67	-9.1E+00	-8.7E+00	6.6E+00	-7.2E+01	-2.3E+01	2.8E+00	6.6E-02
Ozone layer depletion	36.98	-3.1E-08	-2.7E-08	8.0E-08	-2.6E+02	-2.0E-07	1.1E-07	8.0E-10
Respiratory inorganics	18.67	-6.2E-04	-5.8E-04	6.9E-04	-1.1E+02	-2.0E-03	6.4E-04	6.9E-06
Respiratory organics	34.29	-3.4E-04	-2.5E-04	6.4E-04	-1.9E+02	-1.7E-03	7.2E-04	6.4E-06
Terrestrial acid/nutri	33.33	-7.2E-03	-5.9E-03	1.4E-02	-2.0E+02	-3.7E-02	1.8E-02	1.4E-04
Terrestrial ecotoxicity	47.54	-1.6E+00	-1.4E+00	2.3E+01	-1.5E+03	-4.8E+01	4.3E+01	2.3E-01
Confidence interval:	95							

Table S6

Uncertainty analysis results for comparison between S-2 and S-3, midpoint,1 p 'S-3' (A) minus 1 p 'S-2' (B).

Impact category	A >= B(%)	Mean	Median	SD	CV	2.5%	97.5%	SEM
Aquatic acidification	0.0E+00	-7.7E-04	-7.7E-04	3.1E-05	-4.0E+00	-8.3E-04	-7.1E-04	3.1E-07
Aquatic ecotoxicity	0.0E+00	-1.6E+01	-1.6E+01	4.3E-01	-2.8E+00	-1.6E+01	-1.5E+01	4.3E-03
Aquatic eutrophication	0.0E+00	-3.9E-05	-3.9E-05	1.0E-06	-2.6E+00	-4.1E-05	-3.7E-05	1.0E-08
Carcinogens	0.0E+00	-2.4E-02	-2.4E-02	5.0E-04	-2.1E+00	-2.5E-02	-2.3E-02	5.0E-06
Global warming	0.0E+00	-1.3E-01	-1.3E-01	5.2E-03	-4.1E+00	-1.4E-01	-1.2E-01	5.2E-05
Ionizing radiation	0.0E+00	-1.3E+01	-1.3E+01	2.9E-01	-2.2E+00	-1.4E+01	-1.3E+01	2.9E-03
Land occupation	0.0E+00	-6.2E-03	-6.2E-03	1.4E-04	-2.3E+00	-6.4E-03	-5.9E-03	1.4E-06
Mineral extraction	0.0E+00	-4.1E-02	-4.2E-02	8.6E-04	-2.1E+00	-4.3E-02	-4.0E-02	8.6E-06
Non-carcinogens	0.0E+00	-7.1E-03	-7.1E-03	1.6E-04	-2.2E+00	-7.4E-03	-6.8E-03	1.6E-06
Non-renewable energy	0.0E+00	-3.4E+00	-3.4E+00	1.1E-01	-3.2E+00	-3.7E+00	-3.2E+00	1.1E-03
Ozone layer depletion	1.0E+02	1.4E-08	1.4E-08	1.8E-10	1.3E+00	1.4E-08	1.5E-08	1.8E-12
Respiratory inorganics	0.0E+00	-2.2E-04	-2.2E-04	8.4E-06	-3.8E+00	-2.4E-04	-2.0E-04	8.4E-08
Respiratory organics	1.0E+02	1.4E-04	1.4E-04	2.1E-06	1.5E+00	1.4E-04	1.4E-04	2.1E-08
Terrestrial acid/nutri	1.0E+02	3.8E-04	3.8E-04	8.9E-05	2.4E+01	2.0E-04	5.5E-04	8.9E-07
Terrestrial ecotoxicity	0.0E+00	-4.3E+00	-4.3E+00	1.2E-01	-2.8E+00	-4.5E+00	-4.1E+00	1.2E-03
Confidence interval:	95							

Table S7

Uncertainty analysis results for comparison between S-1 and S-2, endpoint,1 p 'S-2' (A) minus 1 p 'S-1' (B).

Damage category	A >= B (%)	Mean	Median	SD	CV	2.5%	97.5%	SEM
Climate change	18.94	-3.73E-01	-3.45E-01	4.05E-01	-1.08E+02	-1.20E+00	3.45E-01	4.05E-03
Ecosystem quality	66.3	2.10E-01	2.08E-01	4.73E-01	2.25E+02	-7.01E-01	1.12E+00	4.73E-03
Human health	27.74	-3.35E-07	-3.19E-07	5.25E-07	-1.57E+02	-1.38E-06	6.35E-07	5.25E-09
Resources	19.91	-5.82E+00	-5.46E+00	6.59E+00	-1.13E+02	-1.92E+01	6.03E+00	6.59E-02
Confidence interval:	95							

Table S8

Uncertainty analysis results for comparison between S-1 and S-3, endpoint,1 p 'S-3' (A) minus 1 p 'S-1' (B).

Damage category	A >= B(%)	Mean	Median	SD	CV	2.5%	97.5%	SEM
Climate change	10.35	-4.9E-01	-4.6E-01	4.0E-01	-8.2E+01	-1.3E+00	2.3E-01	4.0E-03
Ecosystem quality	63.51	1.7E-01	1.7E-01	4.8E-01	2.8E+02	-7.7E-01	1.1E+00	4.8E-03
Human health	13.77	-5.7E-07	-5.4E-07	5.3E-07	-9.2E+01	-1.6E-06	4.0E-07	5.3E-09
Resources	7.49	-9.2E+00	-8.8E+00	6.6E+00	-7.2E+01	-2.3E+01	2.7E+00	6.6E-02
Confidence interval:	95							

Table S9

Uncertainty analysis results for comparison between S-2 and S-3, endpoint,1 p 'S-3' (A) minus 1 p 'S-2' (B).

Damage category	A >= B(%)	Mean	Median	SD	CV	2.5%	97.5%	SEM
Climate change	0	-1.3E-01	-1.3E-01	5.2E-03	-4.1E+00	-1.4E-01	-1.2E-01	5.2E-05
Ecosystem quality	0	-4.1E-02	-4.1E-02	1.2E-03	-3.0E+00	-4.3E-02	-3.9E-02	1.2E-05
Human health	0	-2.4E-07	-2.4E-07	7.8E-09	-3.2E+00	-2.6E-07	-2.3E-07	7.8E-11
Resources	0	-3.5E+00	-3.5E+00	1.1E-01	-3.2E+00	-3.7E+00	-3.3E+00	1.1E-03
Confidence interval:	95							

Table S10

Uncertainty analysis results for comparison between S-1 and S-2, Single score,1 p 'S-2' (A) minus 1 p 'S-1' (B).

Damage category	A >= B (%)	Mean	Median	SD	CV	2.5%	97.5%	SEM
Single score	29.43	-1.08E-04	-1.03E-04	1.85E-04	-1.72E+02	-4.78E-04	2.38E-04	1.85E-06
Confidence interval:	95							

Table S11

Uncertainty analysis results for comparison between S-1 and S-3, Single score,1 p 'S-3' (A) minus 1 p 'S-1' (B).

Damage category	A >= B(%)	Mean	Median	SD	CV	2.5%	97.5%	SEM
Single score	17.15	-1.8E-04	-1.7E-04	1.9E-04	-1.0E+02	-5.6E-04	1.7E-04	1.9E-06
Confidence interval:	95							

Table S12

Uncertainty analysis results for comparison between S-2 and S-3, Single score,1 p 'S-3' (A) minus 1 p 'S-2' (B).

Damage category	A >= B(%)	Mean	Median	SD	CV	2.5%	97.5%	SEM
Single score	0	-7.3E-05	-7.3E-05	2.5E-06	-3.3E+00	-7.8E-05	-6.9E-05	2.5E-08
Confidence interval:	95							
